# Estimating the cost of training disruptions on marathon performance

**DOI:** 10.3389/fspor.2022.1096124

**Published:** 2023-01-10

**Authors:** Ciara Feely, Barry Smyth, Brian Caulfield, Aonghus Lawlor

**Affiliations:** ^^1^^ SFI Center for Research Training in Machine Learning, Dublin, Ireland; ^^2^^ Insight Center for Data Analytics, University College Dublin, Dublin, Ireland

**Keywords:** marathon running, marathon training, running related injuries, marathon, recreational runners, recreational running, marathon performance, data analysis

## Abstract

Completing a marathon usually requires at least 12–16 weeks of consistent training, but busy lifestyles, illness or injury, and motivational issues can all conspire to disrupt training. This study aims to investigate the frequency and performance cost of training disruptions, especially among recreational runners. Using more than 15 million activities, from 300,000 recreational runners who completed marathons during 2014–2017, we identified periods of varying durations up to 16 weeks before the marathon where runners experienced a complete cessation of training (so-called training disruptions). We identified runners who had completed multiple marathons including: (i) at least one disrupted marathon with a long training disruption of ≥7 days; and (ii) at least one undisrupted marathon with no training disruptions. Next, we calculated the performance cost of long training disruptions as the percentage difference between these disrupted and undisrupted marathon times, comparing the frequency and cost of training disruptions according to the sex, age, and ability of runner, and whether the disruptions occurred early or late in training. Over 50% of runners experienced short training disruptions up to and including 6 days, but longer disruptions were found to be increasingly less frequent among those who made it to race-day. Runners who experience longer training disruptions (≥7 days) suffer a finish-time cost of 5–8% compared to when the same runners experienced only short training disruptions (<7 days). While we found little difference (<5%) in the likelihood of disruptions—when comparing runners based on sex, age, ability, and the timing of a disruption—we did find significant differences in the the cost of disruptions (10–15%) among these groups. Two sample t-tests indicate that long training disruptions lead to a greater finish-time cost for males (5%) than females (3.5%). Faster runners also experience a greater finish-time cost (5.4%) than slower runners (2.6%). And, when disruptions occur late in training (close to race-day), they are associated with a greater finish-time cost (5.2%) than similar disruptions occurring earlier in training (4.4%). By parameterising and quantifying the cost of training disruptions, this work can help runners and coaches to better understand the relationship between training consistency and marathon performance. This has the potential to help them to better evaluate disruption risk during training and to plan for race-day more appropriately when disruptions do occur.

## 1. Introduction

Running is a popular form of exercise and globally there has been a notable increase in the number of recreational runners who wish to test their limits by training for, and participating in, endurance events such as the marathon (1, 2). Indeed big city marathons routinely attract tens of thousands of participants and hundreds of thousands of spectators, making these races among the largest sporting events in the world (3). Competing in a marathon typically requires an extended period (12–16 weeks) of dedicated training, with runners usually following prescribed training programmes to gradually build their endurance, strength and speed (4, 5).

Marathon training programmes are designed to be periodic in their structure (4, 6), with progressive weeks of increasing training load punctuated by lighter weeks to allow runners to recover from, and adapt to, the stresses of training. For example, programmes will often implement 4-week cycles of training and recovery, with an 3-week period of increasing training load following by a 1-week recovery period with less training intensity before moving on to the next 4-week block. As such a typical marathon programme will cover 3–4 blocks of training with training load usually peaking 4–6 weeks before race-day. Most programmes also incorporate a gradual reduction in training load in the 2–3 weeks directly before race-day—the so-called taper period—so that the runner can recover fully for their race (7–10). These programmes are designed to allow runners to gradually improve their endurance and strength by carefully increasing training load in a manner that is safe and effective. Programmes rarely incorporate any extended periods without any training, and maintaining a consistent training schedule is usually recommended for optimal performance (11). However, marathon training imposes a considerable physical and psychological burden on runners and many become injured or take breaks from regular training for a variety of reasons (12). At best this can disrupt training for an extended period of time, but it may even prevent runners from making it to race-day. While there have been a number of studies on the relationship between various training factors (e.g. long runs, weekly distance, average pace etc.) (11), and the effect of periods of de-training on runner fitness (13), less is known about the frequency of training disruptions and their impact on marathon performance, especially among recreational runners.

In this paper we are interested in the important of training consistency and the cost of disruptions and our focus on recreational runners is born out of a desire to understand these issues in the context of this very large group of runners where such questions routinely arise. We investigate cost of training disruptions on marathon performance for recreational runners, using training data from 292,323 recreational runners who completed marathons during 2014–2017. These runners were all users of the popular running app, Strava, and this work is an example of the new type of data-driven research that has been enabled by the availability of wearable technology, body sensors, and smart watches/phones, which are now commonly used to record the exercise and activity habits of millions of people around the world. Indeed the availability of similar datasets has already facilitated a number of large-scale studies of marathon training and performance (14–16), and even offered the opportunity to develop personalised training recommendations for marathon runners (17–20).

Even the most determined runners can struggle to achieve the right work-life-training balance when training for the marathon, and busy lifestyles, work commitments, illness and injury can all conspire to disrupt training. However, relatively little is known about the prevalence and patterns of disruptions in the training programmes of recreational runners. The novel contribution of this work stems from its focus on training disruptions—extended periods where a runner performs no training—to evaluate their frequency among recreational runners and their finish-time cost on race-day. We further consider how the frequency and cost of disruptions vary depending according to runner sex, age, ability and the timing of disruptions (whether early or late in training).

## 2. Materials and methods

### 2.1. Subjects and data

This work is based on a dataset made available to the authors via a data sharing agreement with the popular mobile fitness application Strava. The data used in this study comprises 15,697,711 individual running activities recorded by 292,323 individual runners who completed 509,979 marathons during the period 2014–2017; see Table 1 and the discussion that follows. It should be noted that this dataset only includes runners who participated in at least one marathon event during the 2014–2017 period and, therefore, it does not contain data for runners who may have intended to participate in marathons but who were unable to, because of injury, illness, or for some other reason; it was reported in 1987 that approximately 16% of runners who signed up for a marathon failed to participate (21). We discuss the implications of this later in this paper.

**Table 1:** A summary of the dataset used in this work showing for male and female runners, the number of unique runners, their mean age and marathon times, the number of marathons completed in the period, and their average distance per week and number of activities per week during training.

Sex	Unique runners	Age	MT	No. races	Dist/Wk	Day/Wk
F	59,118	38.51	264.23	1.57	40.56	3.12
M	233,205	40.22	239.85	1.79	41.8	3.07

#### 2.1.1. Activities

The set of activities of a runner r are indicated as A(r) in Equation 1. Each activity $a_{i}$ comprises a set of distances and times sampled at 100 m intervals from the raw GPS data uploaded to Strava. From these data we calculate the runner’s mean pace for each 100 m interval. Each activity is also associated with the date and time when the activity occurred.

(1) $$A(r)=\{a_{1},\ldots ,a_{n}\}$$

An activity is deemed to be a marathon if the total distance completed ($D(a_{i})$) is between 42,195 m ±5% as shown in Equation 2.

(2) $$\text{Marathon}(a_{i})\;⟺\;40,085\leq D(a_{i})\leq 44,305$$

#### 2.1.2. Marathon races

The finish-time of each marathon activity is an estimate of the time it took the runner to complete the full marathon distance (42,195 m). This means that the finish-times of slightly shorter or longer activities are scaled accordingly, based on the average pace of the activity up to 42,195 m. To avoid issues with mislabelled activities (e.g. cycling as running) or GPS errors all marathon activities must be between 2 and 7 h in duration and those that are shorter or faster are excluded.

Obviously, the above approach is not guaranteed to identify activities that correspond to organised marathon races; some runners may run marathon length sessions during training. To account for this, in our dataset we identify each runner’s fastest marathon-length activity during the Spring (January–June) and Autumn (July–December) marathon seasons each year; see Equation 3 where P refers to the activities for a given period (Spring/Autumn and year).

(3) $$\text{Fastest}(r,P)=a_{p}|\text{Marathon}(a_{p})\;\&\;\;{}_{a_{i}\in P}\forall \;\;\;\;\;\text{pace}(a_{p})<\;\text{pace}(a_{i})$$

#### 2.1.3. Marathon training

Each fastest marathon is then associated with the runner’s training activities during the 16-week period directly before this fastest marathon, and these activities are taken to be the runner’s training for this fastest marathon; see Equation 4.

(4) $$T(r,P)=\{a_{i}\in A(r)|\text{date}(\text{Fastest}(r,P))-\text{date}(a_{i})\leq 16\times 7\;\text{days}\}$$

For this work, we further ensured that each set of marathon training activities contained at least 10 weeks of training data and at least 20 days of training activities. The resulting dataset included 15,697,711 individual activities in 509,979 such training sets for 292,323 unique runners (80% male and 20% female). This dataset is summarised in Table 1.

#### 2.1.4. Runner ability

To estimate a runner’s ability we use their overall fastest marathon finish-time as a proxy for their personal-best (PB) time, during the 2014–2017 period of the dataset. This allows us to distinguish between two distinct standards: (i) fast runners with a PB time less than 240 min (4 h); and (ii) slow runners with a PB time greater than 240 min. We choose 240 min because it is an iconic finish-time for the marathon among recreational runners (22). In our dataset 157,050 runners are fast by this definition (86.62%:13.38% male:female) and 135,273 runners are slow (71.83%:28.17% male:female).

### 2.2. Defining training disruptions

A training disruption is defined as a consecutive sequence of at least n days without any logged training activities. Few runners will train every day of the week. Most will train for 3–4 days per week. Therefore, sequences of 1 or 2 days without training are to be expected. Many runners will also take short breaks in their training, perhaps up to a week in duration, maybe due to a busy lifestyle or a lack of motivation, or perhaps a short illness. However, longer disruptions are more likely to be associated with illness or injury or some other event.

#### 2.2.1. Longest training disruptions (LTD)

For each training set, T=Training(r,P), we define the maximum/longest disruption length (longest training disruption or LTD) according to Equation 5. In this way we can associate each training set with a maximum number of consecutive days during which no training occurred.

(5) $$\mathrm{LTD}(T)=\max_{a_{i}\in T}\text{date}(a_{i+1})-\text{date}(a_{i})$$

#### 2.2.2. Late versus early disruptions

Furthermore, we say that the longest training disruption is early if it occurs during the early weeks of training (8–12 weeks from race-day), and it is late if it occurs later in training (3–7 weeks from race-day); note that we do not include the final three weeks of training because this is the taper period when runners tend to reduce their training (7).

#### 2.2.3. Disrupted versus undisrupted training

The main objective of this work is to estimate the finish-time cost of training disruptions by estimating how finish-times change as a result of disruptions. To do this we define a training set (T) to be disrupted if the training period 3–12 weeks before race-day is associated with a longest training disruption of at least 7 days as shown in Equation 6. Likewise, we say that a training set is undisrupted if it has a longest training disruption strictly less than 7 days. Without loss of generality, in what follows we will refer to disrupted/undisrupted marathon as a marathon that is associated with a disrupted/undisrupted set of training activities.

(6) Disrupted(T)⟺LTD((T))≥7

(7) Undisrupted(T)⟺LTD(T)<7

#### 2.2.4. Disruption cost

To compute an estimate of the disruption cost we compare race-day finish-times of disrupted and undisrupted marathons on a per-runner basis. That is, we focus on runners who have at least one disrupted and at least one undisrupted marathon, such that the time between the disrupted and undisrupted marathons is at least 6 months and no more than 2 years. There are 43,933 unique such runners (83.39% male and 26.61% female) producing 56,735 pairs of disrupted/undisrupted training sets; some runners have more than one disrupted and undisrupted marathons.

The disruption cost is estimated based on the relative difference between the disrupted and undisrupted marathon finish-times, as shown in Equation 8, where $FT(D_{r})$ and $FT(U_{r})$ denote the finish-times of the disrupted and undisrupted marathons for runner, r, respectively.

(8) $$\mathrm{Cost}(D_{r},U_{r})=\frac{FT(D_{r})}{FT(U_{r})}-1$$

For example, if a runner completes a disrupted marathon—that is, a marathon for which their training included a ≥7 day break—in 244 min compared to a recent time of 235 min for an undisrupted marathon, then the disruption cost is 0.038 (=9/235a, or a 3.8% slowdown). In this work we consider how this disruption cost varies with runner sex, age, ability, the timing of the disruption (early versus late) and the duration of the disruption.

### 2.3. Statistical methods

We analyse the mean disruption cost, by comparing the runners based on sex, age, timing, and ability, using a Tukey test at the 99% confidence level to confirm the existence of significant differences in cost when we compare runners for different disruption durations, 7–13 days, 14–20 days, 21–27 days, and 28 days or more. This is followed by a standard t test to evaluate the significance of pairs of differences within/between groups. A two proportion z-test at the 1% level of significance is used when comparing the proportions of runners within and across groups. For each test, the effect size was measured using Cohen’s d.

## 3. Results

### 3.1. Marathon training disruption frequency

**Figure 1:**
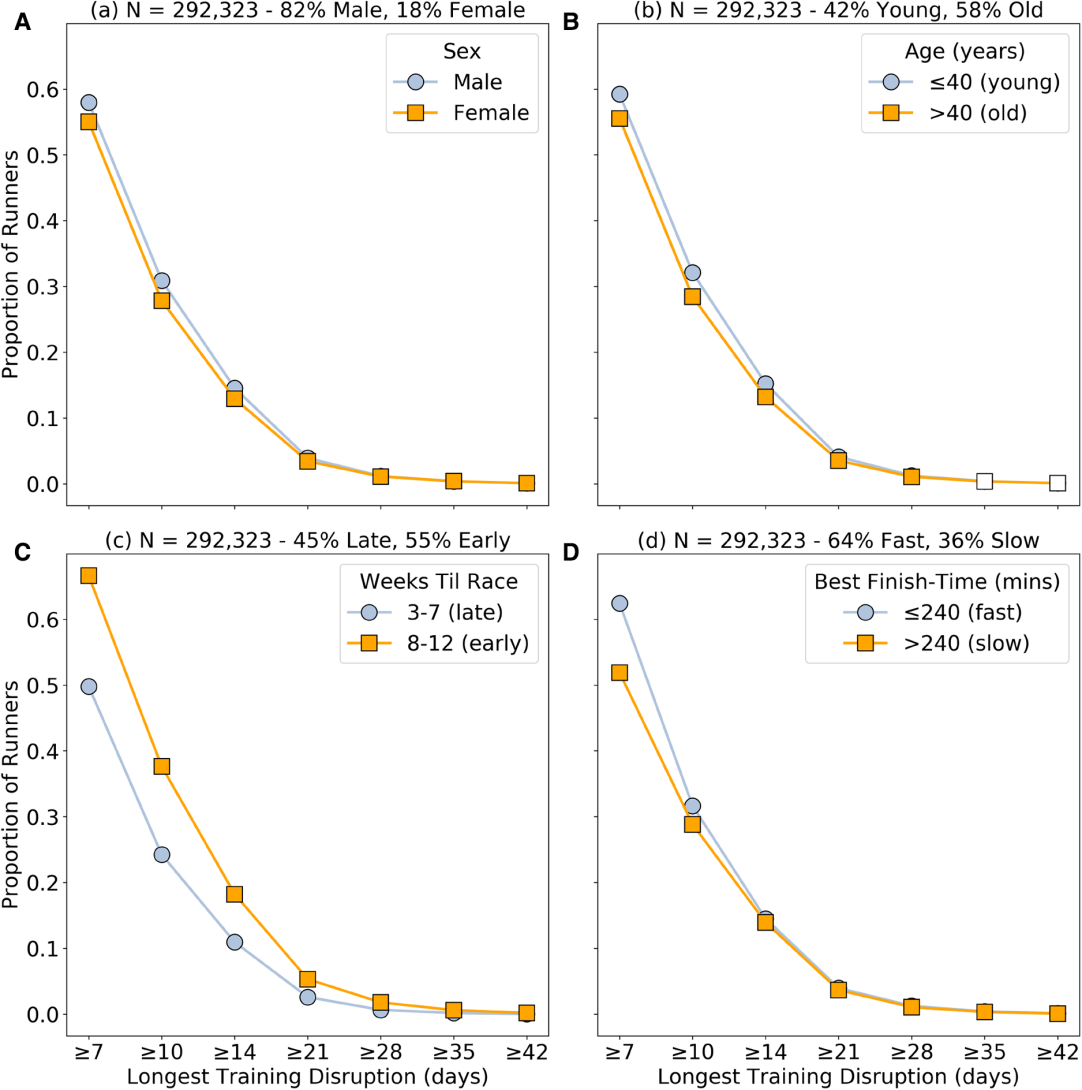
The cumulative proportion of runners experiencing progressively longer maximum training disruptions (≥7, 10, 14, 21, 28, 35, 42 days) based on: (**A**) sex (male versus female); (**B**) age (≤40 years-old vs >40 years-old); (**C**) timing (early or late in training); and (**D**) runner ability (based on whether their fastest marathon was faster, ≤240 min, or slower, ≥240 min). The statistical significance of the difference in proportions between each group, for a given longest disruption length, is calculated based on a two-proportion z-test with p<0.01 and indicated by filled (p<0.01) or unfilled (p≥0.01) markers.

Figure 1 shows the proportion of runners experiencing progressively longer training disruptions (LTD as per Equation 5), comparing runners based on sex, age, disruption timing, and ability. For example, approximately 58% of male runners experience a training disruption of at least 7 days, compared to 55% of female runners; see Figure 1A; which is a statistically significant difference (p<0.01). A measure of effect size was also calculated using Cohen’s d, and found to be >1 for all statistically significant differences presented in this section, indicating a large effect size in all cases. In this graph statistical significance is denoted by a colored in marker. Figures 1A,C,D have statistically significant differences for all disruption durations, whereas Figure 1B shows insignificant differences for the longer disruption durations.

### 3.2. The cost of training disruptions

**Figure 2:**
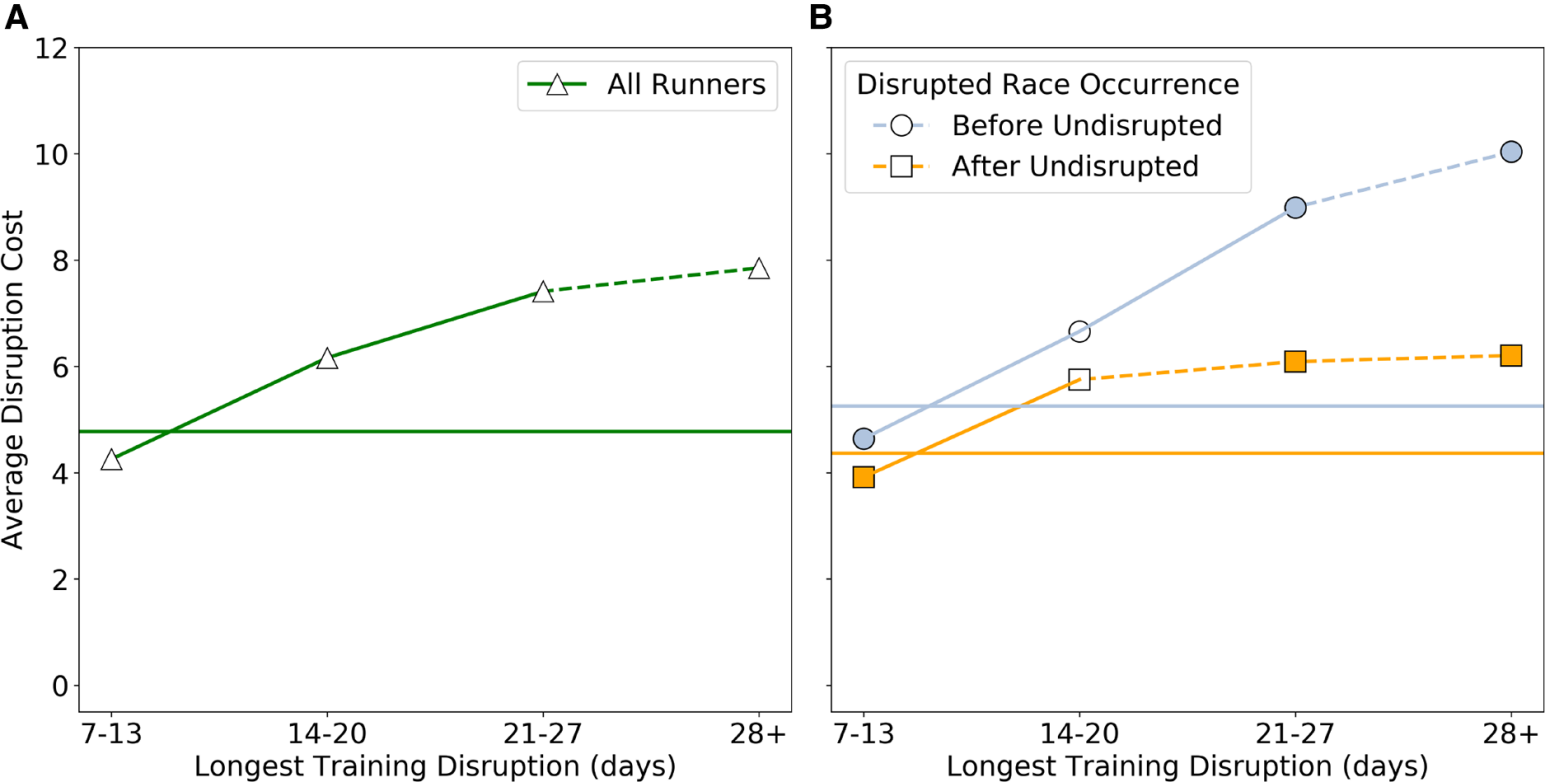
(**A**) The average disruption cost of all runners, based on the difference in marathon time for disrupted and undisrupted training periods, and the length of the longest training disruption in days. (**B**) The average disruption cost depending on whether the disrupted training programme occurs before (blue) or after (orange) the undisrupted training programme. In both (**A**) and (**B**) a solid line between markers indicates that the difference between consecutive durations of training disruptions is statistically significant based on a t test with p<0.01. In (**B**) a filled in marker indicates for a given disruption duration a statistically significant (based on a t-test with p<0.01) difference between the before and after groups.

Figure 2 shows the disruption cost versus the length of the longest disruption; disruption cost is calculated as the average percentage difference in finish-time between disrupted and undisrupted races as defined in Equation 8 in Section 2. For example, on average, runners who experience a 7–13 day longest training disruption experience a 4.25% increase in their marathon finish times, compared to races where the same runners do not experience a training disruption that is longer than 6 days.

In turn, Figure 2B shows a modified version of the disruption cost to distinguish between runner pairs in which the disrupted race occurred before or after the undisrupted race.

**Figure 3:**
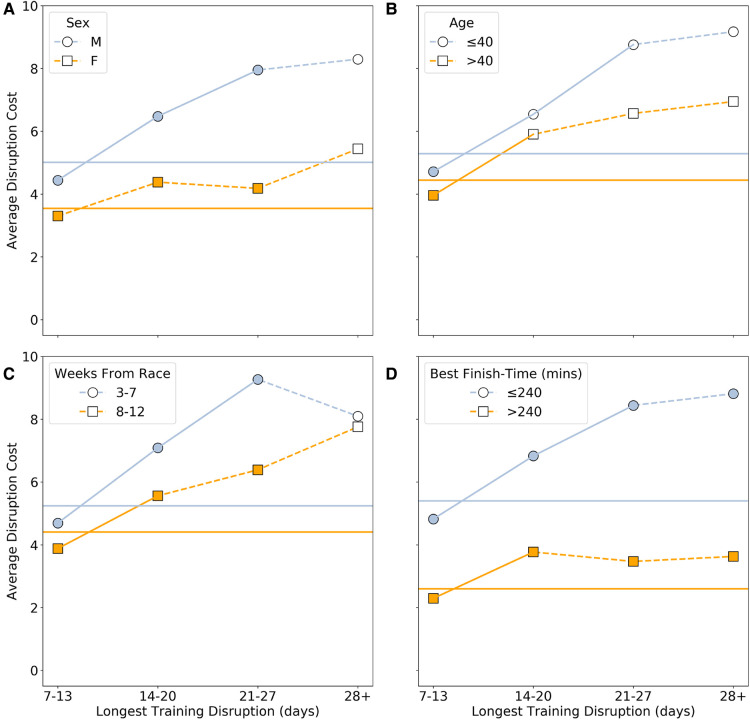
The average disruption cost based on: (**A**) sex (male versus female); (**B**) age (≤40 years-old vs >40 years-old); (**C**) timing (early or late in training); and (**D**) runner ability (based on whether their fastest marathon was faster, ≤240 min, or slower, ≥240 min). Statistical significance between groups, for a given disruption duration, is calculated based on a t-test with p<0.01 and indicated by filled (p<0.01) or unfilled (p≥0.01) markers. The within-group statistical significance of differences between consecutive disruption durations is calculated using a t-test with p<0.01 and indicated by solid (p<0.01) or dashed (p≥0.01) connecting lines. The horizontal lines indicate the mean values for each pair of groups; if the difference between the overall means is significant then the lines are solid otherwise they are dashed.

Next, Figure 3 shows how this relationship between disruption cost and duration varies based on runner sex (a), age (b), disruption timing (c), and runner ability (d).

For completeness, Table 2 shows the number of runner pairs associated with each longest training disruption (LTD) category used in the disruption cost calculations; each pair corresponds to a single runner with an undisrupted and disrupted training history. In addition, the percentages of pairs based on sex, age, timing, and ability are also show.

**Table 2:** Number of race-pairs for different max disruption lengths (N) broken down for sex, age, timing and ability. Here WFR denotes weeks-from-race-day, DRD refers to disrupted race date and UDR refers to undisrupted race date.

LTD	No. pairs	%DRD<URD	%Sex=M	%Age≤40	%WFR<7	%MT≤240
7–13	43,960	53.38	83.77	39.16	46.01	77.57
14–20	9,064	54.74	85.02	40.31	39.35	78.13
21–27	2,411	54.42	85.69	38.45	35.50	79.30
28+	1,300	57.00	84.46	40.69	27.84	81.46

## 4. Discussion

In Figure 1 we see that although training disruptions of at least 7 days are experienced by more than 50% of runners, progressively longer disruptions are increasingly less common; regardless of sex, age, timing or ability, the frequency of longer disruptions falls considerably. The differences based on sex, age and ability are much less pronounced (≤10% between groups)—see Figures 1A,B,D—than the difference for the early versus late disruptions (≈15–25%) shown in Figure 1C. In other words, male runners, younger runners, or faster runners are more likely to experience disruptions of a given duration compared with females, older runners, or slower runners respectively, but when disruptions do occur they are more likely to occur earlier in training. For all sitatistically significant differences, denoted by a filled in marker, and calculated based on a two-proportion z-test with p<0.01, the effect size Cohen’s d measured an effect size of d>1 which is considered large.

In our dataset we only have access to runners, and their training data, if they made it to race-day. One limitation of this is that the greater proportion of early disruptions may be due to an under-counting of late training disruptions, if a runner with late training disruptions are more likely withdraw from a race, and therefore are not present in our dataset. Since longer disruptions are more likely to be associated with injury or illness, it is reasonable to expect late training disruptions to be associated with higher withdrawal rates, because runners will have less time to recover before race-day. This is consistent with the results of Clough et al. (21) which found that over 50% of runners who withdrew from one Scottish marathon cited injury or illness as the reason for their withdrawal. Thus, it appears likely that the larger differences in the proportion of runners experiencing early and late training disruptions may be an artefact of the dataset rather than an increased likelihood of disruption earlier in training. If this is indeed the case, then we can conclude, based on the results presented in Figure 1 that there are only modest differences in the likelihood of runners experiencing training disruptions based on sex, age, timing and ability.

While it is not uncommon for runners to experience some material disruption (7–14 days and greater) in their training, the central question for this work concerns the future finish-time cost of such disruptions when they occur. Figure 2A indicates that the disruption cost increases with the degree or duration of disruption. This might be expected for at least two reasons. First, longer breaks from training are more likely to result in detraining and reduced fitness; the work of Chen et al. (13) reported a significant decrease in key fitness metrics as a result of just two weeks of detraining among male endurance athletes. Second, if the absence from training was due to injury then the runner may need to take longer to return to their training trajectory, if they do at all.

It is also worth noting that this increasing cost occurs regardless of whether the disrupted race took place before or after the undisrupted race used as the basis for the cost calculation; see Figure 2B. The lower cost when the disrupted race occurs after the undisrupted race may be due to a combination of age and experience related effects. On the one hand, as runners get older (>30 years old) their marathon finish-times tend to increase. On the other hand, more experience helps to moderate age these age related effects. Either way, Figure 2B indicates that longer training disruptions tend to be associated with greater disruption costs regardless of the order of disrupted and undisrupted races used as the basis for the disruption cost calculation.

A similar trend is evident when we compare disruption costs based on runner sex, age, timing and ability, as shown in Figures 3A–D. In each case the mean disruption cost for males, younger runners, late disruptions, and faster runners is significantly greater than that for females, older runners, early disruptions and slower runners, based on a t test with p<0.01, and as indicated by the horizontal lines in Figures 3A–D. However, there is some variation in the significance of the changes in disruption cost by disruption duration across the various groups. For all statistically significant values, Cohen’s measure of effect size d was large, with values greater than 1.

For example, the within-group differences in disruption cost, between consecutive disruption duration categories, are not always significant, but the between-group differences usually are, at least up to 21–27 day disruptions. Even though similar proportions of male and female runners experience disruptions of a given duration—Figure 1A—the disruption cost for males is significantly greater than the disruption cost for females for a given disruption duration, except for ≥28 day disruptions; see Figure 3A. Thus, a male runner with a 21–27 day training disruption can expect to experience close to an 8% increase in their finish-time compared to their corresponding undisrupted race, but for similarly disrupted female runners, the cost is just over 4%. One reason for this sex difference may that male runners tend to overestimate their capability (23) and tend to run less disciplined races (24, 25, 14), which may compound the effects of training disruptions on race-day.

Differences based on the timing of disruptions show some of the greatest disruption costs. For example, those experiencing a 21–27 day disruption in the 3–7 weeks before race-day can expect to suffer an almost 10% increase in their finish-times, all other things being equal, compared to just 6% when a similar length duration occurs much earlier in training. Obviously, the earlier disruption affords the runner much more time to recover from any detraining that may have occurred, and from injury if that was the cause of the disruption. In contrast, a long break in the weeks before race-day provide little time for a return to form. Indeed, as before, it is also worth point out that the disruption cost associated with these late training disruptions likely underestimate the true cost of such disruptions, since at least some runners can be expected to withdraw entirely from the race.

The greatest disruption cost differences occur between fast and slow runners; the former are associated with a disruption cost that is 2–3x as great as the latter. This may be due in part to male runners that are associated with greater disruption cost making up a larger proportion (88%) of the faster group.

Finally, it is worth noting some limititations of this study. The data we have contains only the training sessions logged into Strava, and we have no contact with the runners who log them. This means that we don’t know if a training disruption is a true disruption, or simply a period in which runners trained without logging their sessions into Strava. Additionally, not having access to the runners means that we don’t know the reason for the training disruption—whether it was due to illness, injury, demotivation, or life getting in the way. In particular, understanding which disruptions are due to injury would allow for more detailed study of performance cost.

## 5. Conclusions

The central contribution of this study is an analysis of the frequency and performance implications of disruptions in the training of recreational marathon runners. Based on an analysis of 292,323 runners who competed in 509,979 marathons between 2014–2017, we found that a majority of runners experienced training disruptions of at least 7 days at some stage during training, but that progressively longer disruptions were increasingly less likely; the frequency of disruptions was similar regardless of runner sex, age, ability or when the disruption occurred. We estimated the cost of training disruptions by comparing the race-times of runners with disrupted training to the race-times of the same runners without training disruptions and found a significant increase in finish-times (2–9%) depending on runner age, sex, ability, and the timing and duration of disruption. Male runners, younger runners, and faster runners all suffered greater disruption costs than female, older, or slower runners, and disruptions that occurred later in training were more costly than those occurring early in training.

By parameterising and quantifying the cost of training disruptions this work will help runners and coaches to better calibrate their training and race-day expectations, if and when disruptions occur. Moreover, the findings may help to guide future research on the relationship between training, recovery, and injury, in order to, for example, better predict over-training or injury risk, and better support runners as they return from a period of disrupted training.

## Data Availability

The data analyzed in this study is subject to the following licenses/restrictions: Data was obtained through data sharing agreement with mobile application Strava. Other researchers could request access from Strava if they should like to access the data. Requests to access these datasets should be directed to Strava Strava.com.
